# Soluble guanylate cyclase activator BAY 54–6544 improves vasomotor function and survival in an accelerated ageing mouse model

**DOI:** 10.1111/acel.13683

**Published:** 2022-08-27

**Authors:** Ehsan Ataei Ataabadi, Keivan Golshiri, Annika A. Jüttner, René de Vries, Ingrid Van den Berg‐Garrelds, Nicole M. A. Nagtzaam, Hina N. Khan, Frank P. J. Leijten, Renata M. C. Brandt, Willem A. Dik, Ingrid van der Pluijm, A. H. Jan Danser, Peter Sandner, Anton J. M. Roks

**Affiliations:** ^1^ Division of Pharmacology and Vascular Medicine, Department of Internal Medicine Erasmus MC Rotterdam the Netherlands; ^2^ Laboratory Medical Immunology, Department of Immunology Erasmus MC Rotterdam the Netherlands; ^3^ Department of Molecular Genetics Erasmus MC Rotterdam the Netherlands; ^4^ Department of Vascular Surgery Erasmus MC Rotterdam the Netherlands; ^5^ Bayer AG, Pharmaceuticals R&D, Pharma Research Center Wuppertal, Germany & Hannover Medical School Institute of Pharmacology Hannover Germany

**Keywords:** NO‐sGC‐cGMP pathway, oxidative stress, senescence, sGC activation, survival, vascular ageing

## Abstract

DNA damage is a causative factor in ageing of the vasculature and other organs. One of the most important vascular ageing features is reduced nitric oxide (NO)soluble guanylate cyclase (sGC)—cyclic guanosine monophosphate (cGMP) signaling. We hypothesized that the restoration of NO‐sGC‐cGMP signaling with an sGC activator (BAY 54–6544) may have beneficial effects on vascular ageing and premature death in DNA repair‐defective mice undergoing accelerated ageing. Eight weeks of treatment with a non‐pressor dosage of BAY 54–6544 restored the decreased in vivo microvascular cutaneous perfusion in progeroid *Ercc1* ^*∆/−*^ mice to the level of wild‐type mice. In addition, BAY 54–6544 increased survival of *Ercc1* ^*∆/−*^ mice. In isolated *Ercc1* ^*∆/−*^ aorta, the decreased endothelium‐independent vasodilation was restored after chronic BAY 54–6544 treatment. Senescence markers *p16* and *p21*, and markers of inflammation, including *Ccl2, Il6* in aorta and liver, and circulating IL‐6 and TNF‐α were increased in *Ercc1* ^*∆/−*^, which was lowered by the treatment. Expression of antioxidant genes, including *Cyb5r3* and *Nqo1*, was favorably changed by chronic BAY 54–6544 treatment. In summary, BAY 54–6544 treatment improved the vascular function and survival rates in mice with accelerated ageing, which may have implication in prolonging health span in progeria and normal ageing.

## Graphical abstract

DNA repair deficiency in Ercc1∆/− mice cause a disruption of NO‐sGC‐cGMP signaling and a decrease of vasodilator response. These mice die prematurely between the age of 14 and 26 weeks and can be used to test interventions in ageing. Treatment with the BAY 54–6544 improved NO‐mediated vasodilation and attenuated vascular senescence and inflammation markers. Moreover, these changes are not restricted to vascular tissue, but manifest themselves also in liver, an organ that is often used to study changes in common ageing pathways in various models. Thus, BAY 54–6544 could be a potential therapeutic treatment to attenuate vascular ageing and improve survival.

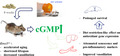

## 1 INTRODUCTION

Cardiovascular diseases are a leading cause of morbidity and mortality worldwide (Gregory et al., 2017). Intrinsic ageing, an independent risk factor of cardiovascular diseases (Lakatta & Levy, 2003), is an inevitable process that is driven by accumulating, non‐repaired DNA damage, which also has implications for cardiovascular function (del Campo et al., 2020; Durik et al., 2012; Kovacic et al., 2011). DNA damage can be induced by different sources like endogenous (e.g. generation of reactive oxygen species [ROS], and other oxidative reactions) or exogenous (e.g. UV and ionizing radiations) reactive agents that may cause hundred thousands of DNA lesions per cell per day which lead to vascular dysfunction and ageing (Incalza et al., 2018). A number of genetically modified mouse strains have been generated that model accelerated ageing based on a specific deficiency in their DNA repair system. Among them, the *Ercc1* ^*∆/−*^ mouse is a convenient model to study vascular ageing. The detailed features of the mouse model have been outlined before (Durik et al., 2012; Weeda et al., 1997). In brief, *Ercc1* ^*∆/−*^ mice have a short life span of around 24–28 weeks and display many human‐like ageing characteristics like neurodegeneration, osteoporosis, liver, kidney, heart, and muscle dysfunction that mostly start from the age of 12 weeks (Durik et al., 2012; Wu et al., 2017) *Ercc1* ^*∆/−*^ mice have been used to study general ageing mechanisms, in which the liver is taken as the organ for target exploration because it is one of the most severely affected organs, and this is believed to contribute to the reduced life span (Durik et al., 2012; Weeda et al., 1997). In addition, the *Ercc1* ^*∆/−*^ mouse is a relevant murine model to study non‐atherosclerotic vascular ageing and mimic key features of non‐atherosclerotic vascular ageing in humans (Bautista‐Nino et al., 2020). Based on conditional, cre‐lox‐mediated gene deletion, endothelium‐specific *Ercc1* knockout mice have been generated by us previously. Also, in these animals, accelerated non‐atherosclerotic vascular ageing is observed, and longevity is reduced to 24–28 weeks (Ehsan Ataei Ataabadi et al., 2021; Bautista‐Nino et al., 2020). Vascular ageing features in *Ercc1* ^*∆/−*^ mice are increased vascular stiffness, decreased vasomotor function, increased wall thickness, and increased cellular senescence. The vasomotor dysfunction involves decreased vasodilation that is mainly mediated by reduced nitric oxide‐soluble guanylate cyclase‐ cyclic guanosine monophosphate (NO‐sGC‐cGMP) signaling pathway, a major player in dysfunction of the aged cardiovascular system (Durik et al., 2012). In the vasculature, NO binds sGC in vascular smooth muscle cells (VSMCs) and generates the second messenger cGMP. Elevated cGMP leads to VSMC relaxation, vasodilation, and reduction in blood pressure. However, cGMP could also have anti‐fibrotic and anti‐remodeling effects (Sandner et al., 2017; Sandner & Stasch, 2017) and might be able to decrease inflammation (Ahluwalia et al., 2004). Due to its abundance throughout the body, the pathogenesis of various diseases has been linked to inappropriate NO‐sGC‐cGMP signaling (Breitenstein et al., 2017; Durgin et al., 2019; Kolijn et al., 2020; Stasch et al., 2002). Interruption of this pivotal signaling pathway could be at least in part due to oxidative stress.

In physiological conditions NO binds to the reduced ferrous (Fe^2+^) heme group in the β‐subunit of sGC, inducing a conformational change of sGC and thereby strongly increasing its cGMP‐production. Under oxidative stress conditions, this Fe^2+^ is oxidized to Fe^3+^, therefore, losing the NO‐binding capacity which stops NO‐sGC binding, activation and cGMP formation (Shah et al., 2018). Persisting oxidative stress finally also leads to heme‐free (apo) sGC which cannot bind NO either (Breitenstein et al., 2017). Recently, it has been shown that an SMC‐specific cytochrome b5 reductase 3 (CYB5R3), is able to reduce sGC back to the native form, further supporting a redox equilibrium in diseases (Rahaman et al., 2017). Given the negative impact of oxidative stress on NO‐sGC‐cGMP signaling previously observed by us and others (Durik et al., 2012), in DNA repair‐deficient accelerated ageing mice, including the *Ercc1* ^*∆/−*^ mouse model, we hypothesized that sGC oxidation might underlie the vasodilator dysfunction in *Ercc1* ^*∆/−*^ mice. If so, cGMP signaling might be restored by using a sGC activator or sGC stimulator. Since sGC activators can activate cGMP production under oxidative stress conditions and independent of NO, whereas stimulators activate non‐oxidized native sGC (Peter Sandner et al., 2021), we have chosen sGC activators for this study. We examined if chronic sGC activator treatment from 8 to 17 weeks of age would attenuate features of vascular ageing and affect longevity in *Ercc1* ^*∆/−*^ mice. Altogether, considering sGC as a key enzyme in NO–cGMP signaling and given the diminished NO‐cGMP function in *Ercc1* ^*∆/−*^, we aimed to study the effect of chronic sGC activation in *Ercc1* ^*∆/−*^ accelerated ageing mice, focusing on changes of NO‐cGMP responsiveness, DNA damage‐related vascular dysfunction, and potential beneficial effects on survival.

## 2 RESULTS

### 2.1 Pilot study

**FIGURE 1:**
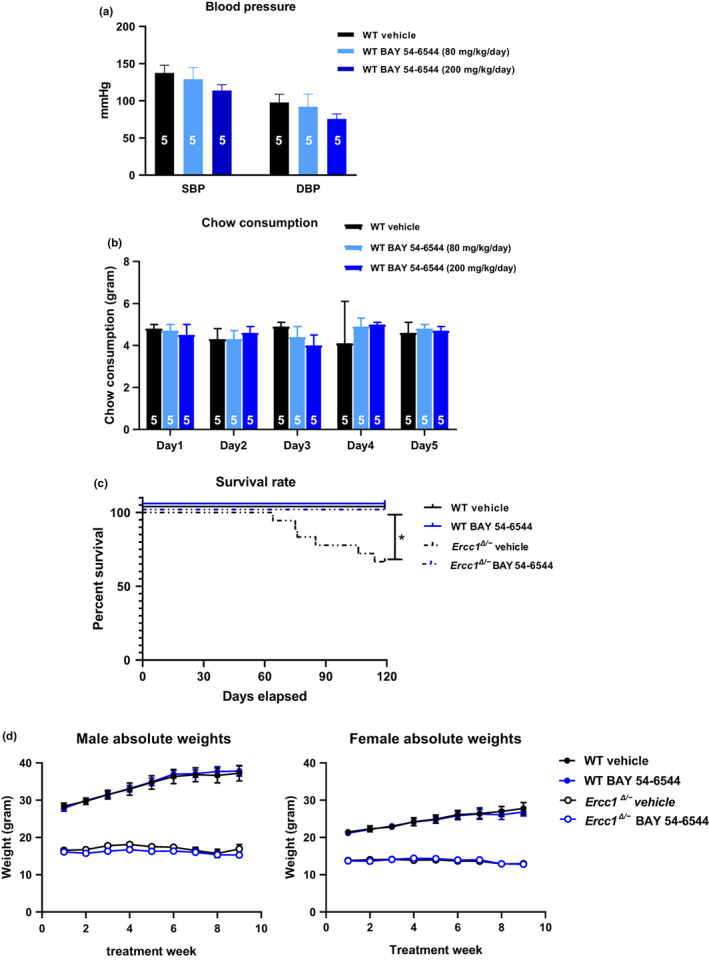
SBP and DBP in mmHg comparison among WT groups treated either with vehicle, low dose (80 mg/kg/day) or high dose (200 mg/kg/day) of the sGC activator BAY 54–6544 (a). The amount of the chow consumption in WT mice receiving either vehicle chow or chow containing 80 mg/kg/day or 200 mg/kg/day of BAY 54–6544 (b). Survival rate at the end of the study in WT and *Ercc1* ^*Δ/−*^ treated either with vehicle or BAY 54–6544 (c). Body weight changes in WT and *Ercc1* ^*Δ/−*^ treated either with vehicle or BAY 54–6544 (200 mg/kg/d) during 8 weeks of treatment (d). Data are presented in mean ± SEM. Statistical differences were analyzed by one‐way ANOVA followed by Dunnett's post hoc test for A and B and log‐rank (Mantel‐Cox) test for C (* = *p* < 0.05). And by general linear model repeated measures for D

For the chronic treatment study, we aimed for a dose‐regimen which have no or very minor effects on systemic arterial blood pressure. We therefore started with a dose‐finding pilot study based on the cardiovascular outcomes in previous studies in rodent models, and measured blood pressure (BP) in wild‐type (WT) mice treated with different chows. WT animals treated for 5 days either with vehicle, low dose (80 mg/kg/day) or high dose (200 mg/kg/day) of BAY 54–6544 were very well tolerated. BAY‐treated mice showed no significant decrease in systolic blood pressure (SBP) and diastolic blood pressure (DBP) for both dosages when compared to control, non‐supplemented chow. However, there was a moderate numerical trend of BP reduction with the high dose which was not significant and did not affect mouse behavior (Figure 1a). We therefore chose the high dose of 200 mg/kg/day for the chronic treatment studies, which warrants the highest drug exposure whilst minimizing the chance of a BP lowering effect. There were no significant differences in food consumption among all the groups (Figure 1b).

### 2.2 Survival rate and body weight

In the vehicle‐treated *Ercc1* ^*Δ/−*^ group, 1/3 of the mice died (6 mice out of 18 died; 33%). Chronic BAY 54–6544 treatment was able to completely prevent mortality in *Ercc1* ^*Δ/−*^ mice (0 mice out of 12 died, 0%) (Figure 1c; *p* < 0.001). All WT mice which served as healthy control, survived. In addition, body weight was preserved during BAY 54–6544 treatment, WT mice gaining weight as expected at this age (Figure 1d).

### 2.3 Blood pressure

**FIGURE 2:**
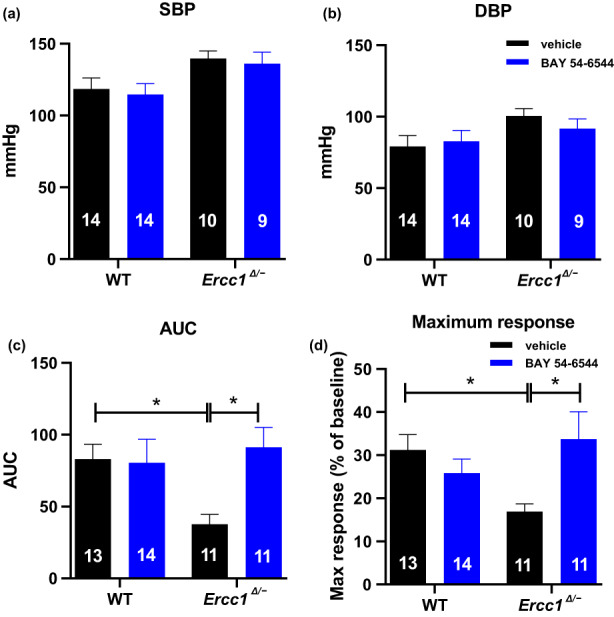
SBP (a) and DBP (b) in mmHg in WT and *Ercc1* ^*Δ/−*^ treated either with vehicle or BAY 54–6544 (200 mg/kg/d). Functional differences between skin reperfusion after 2 minutes of occlusion in calculated area under the curve (AUC) (c) and average maximum response (max response) (d) in WT and *Ercc1* ^*Δ/−*^ treated either with vehicle or BAY 54–6544. Data are presented in mean ± SEM. Statistical differences were analyzed by two‐way ANOVA followed by Bonferroni's post hoc test (* = *p* < 0.05)

No SBP and DBP differences were observed within and between the *Ercc1* ^*Δ/−*^ and WT groups when compared with the vehicle and BAY 54–6544 treated mice (*SBP (mmHg)*: WT vehicle: 118.4 ± 7.8; WT BAY 54–6544: 114 ± 7.5; *Ercc1* ^*Δ/−*^ vehicle: 139.7 ± 5.2; *Ercc1* ^*Δ/−*^ BAY 54–6544:136.1 ± 8.1, *DBP (mmHg)*: WT vehicle: 79.1 ± 7.6; WT BAY 54–6544: 82.7 ± 7.6; *Ercc1* ^*Δ/−*^ vehicle: 100.5 ± 5.1; *Ercc1* ^*Δ/−*^ BAY 54–6544: 91.6 ± 6.8, Figure 2a and b).

### 2.4 Microvascular vasodilator function in vivo

Reactive hyperemia to a 2‐minute hind leg occlusion, measured by laser Doppler indicated by area under the curve (AUC) and the maximum response, was significantly lower in vehicle‐treated *Ercc1* ^*Δ/−*^ than in WT mice (AUC: *p* < 0.03, Maximum response: *p* < 0.03; Figure 2c and d). Treatment with BAY 54–6544 fully normalized both AUC and maximum response as indicators of reactive hyperemia (*AUC* WT vehicle: 31.2 ± 3.6; WT BAY 54–6544: 25.8 ± 3.2; *Ercc1* ^*Δ/−*^ vehicle: 16.9 ± 1.8; *Ercc1* ^*Δ/−*^ BAY 54–6544: 33.7 ± 6.4; *Maximum response*: WT vehicle: 82.9 ± 10.44; WT BAY 54–6544: 80.5 ± 16.4; *Ercc1* ^*Δ/−*^ vehicle: 37.6 ± 6.9; *Ercc1* ^*Δ/−*^ BAY 54–6544: 91.2 ± 13.9. All the values are in arbitrary unit).

### 2.5 Ex vivo vascular function assessment

In organ baths, we tested vasomotor function and mechanisms on isolated aorta. Hereto, concentration response curves (CRCs) of vasorelaxing substances were constructed after preconstricting rings. Preconstriction with 30 nmol/L of U46619 showed a significant reduction in vasoconstriction of both *Ercc1* ^*Δ*/−^ groups compared with WT groups (WT vehicle: 5.36 ± 0.9; WT BAY 54–6544: 5.47 ± 1.4; *Ercc1* ^*Δ/−*^ vehicle: 2.23 ± 0.5; *Ercc1* ^*Δ/−*^ BAY 54–6544: 2.59 ± 0.4. All values are in milli newton [mN]). CRCs were constructed by addition of either endothelium dependent vasodilator acetylcholine (ACh) or endothelium‐independent vasodilators sodium nitroprusside (SNP), sGC stimulator BAY 41–8543, and sGC activator BAY 60–2770 in parallel rings.

**FIGURE 3:**
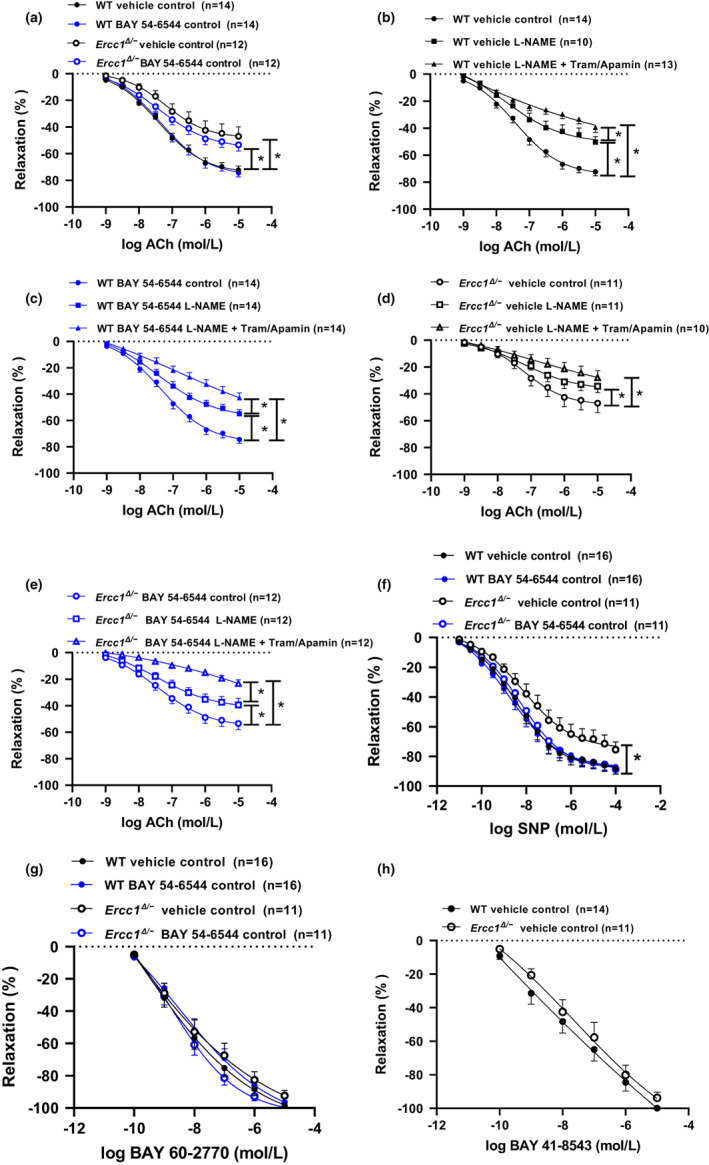
Vasorelaxation (% of preconstriction) in aortic rings of WT and *Ercc1* ^*Δ/−*^ mice treated either with vehicle or BAY 54–6544 in response to ACh (10^−9^ to 10^−5^ Mol/L) (a). The contribution of NO‐cGMP and EDH pathway in WT vehicle (b), WT BAY 54–6544 (c), *Ercc1* ^*Δ/−*^ vehicle (d), and *Ercc1* ^*Δ/−*^ BAY 54–6544 (e) treated mice. Vasorelaxation (% of preconstriction) in aortic rings of WT and *Ercc1* ^*Δ/−*^ mice treated either with vehicle or BAY 54–6544 in response to SNP (10^−11^ to 10^−4^ Mol/L) (f) and sGC activator BAY 60–2770 (10^−10^ to 10^−5^ Mol/L) (g). Vasorelaxation (% of preconstriction) in aortic rings of vehicle‐treated WT and *Ercc1* ^*Δ/−*^ mice in response to sGC stimulator BAY 41–8543 (10^−10^ to 10^−5^ Mol/L) (h). Data are presented in mean ± SEM. Statistical differences were analyzed by general linear model repeated measures for A‐H (* = *p* < 0.05)

The aortic ACh response was lower in vehicle‐treated *Ercc1* ^*Δ*/−^ mice than in the corresponding WT mice. Chronic treatment tended to improve endothelium‐dependent response in *Ercc1* ^*Δ/−*^ mice (Figure 3a; P=NS). Inhibition of endothelial nitric oxide synthase and endothelium‐dependent hyperpolarization (EDH) in aortic segments of both WT groups revealed that the majority of the response to ACh is mediated by NO, while the contribution of EDH was more modest. (Figure 3b and c). In the *Ercc1* ^*Δ/−*^ mice, the NO–cGMP response was smaller (*p* < 0.05, GLM‐RM) than in WT mice (Figure 3d and e). EDH was absent in vehicle‐treated *Ercc1* ^*Δ/−*^ mice (Figure 3d), while BAY 54–6544 treatment restored EDH (Figure 3e).

The endothelium‐independent response to SNP in vehicle‐treated *Ercc1* ^*Δ/−*^ mice was significantly diminished and BAY 54–6544 treatment fully restored the response to the level in WT mice (Figure 3f; *p* < 0.03). The acute endothelium‐independent response to the sGC activator BAY 60–2770 was similar in all groups (Figure 3g). The same was true for the response to the sGC stimulator BAY 41–8543 in vehicle‐treated *Ercc1* ^*Δ/−*^ and WT mice (Figure 3h).

### 2.6 Effects on ageing pathways

**FIGURE 4:**
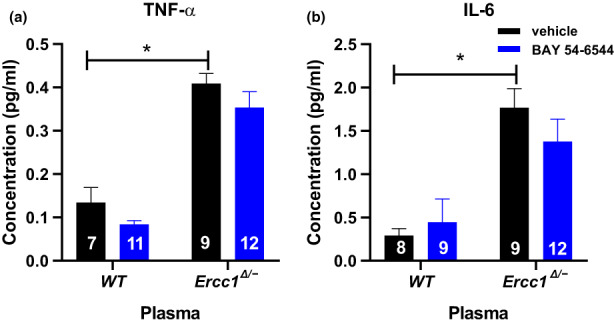
Plasma levels of IL‐6 (pg/ml) and TNF‐alpha in samples in WT and *Ercc1* ^*Δ/−*^ mice treated either with vehicle or BAY 54–6544 for IL‐6 (a) and TNF‐α (b). Data are presented in mean ± SEM. Statistical differences were analyzed by two‐way ANOVA followed by Bonferroni's post hoc test (* = *p* < 0.05).

The improved survival and vascular function prompted us to look further into general ageing features, first focusing on inflammation (Ahluwalia et al., 2004). The plasma levels of TNF‐α and IL‐6 were significantly increased in vehicle‐treated *Ercc1* ^*Δ/−*^ versus corresponding WT mice (Figure 4a and b). BAY 54–6544 treatment in *Ercc1* ^*Δ/−*^ mice modestly reduced cytokine levels, so that the difference versus WT vehicle‐treated mice was no longer significant (Figure 4a and b). Plasma levels of IL‐2, IL‐4, and IFN‐γ were below the lower limit of quantification.

**FIGURE 5:**
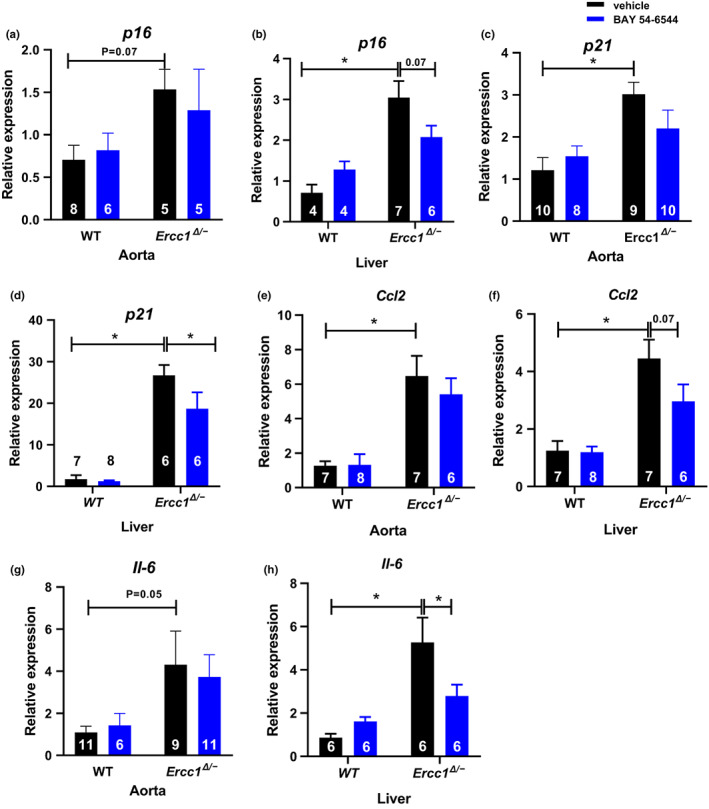
Gene expression of WT and *Ercc1* ^*Δ/−*^ mice treated either with vehicle or BAY 54–6544 for *p16* in aorta (a), *p16* in liver (b), *p21* in aorta (c), *p21* in liver (d), *Ccl2* in aorta (e), *Ccl2* in liver (f), *Il‐6* in aorta (g), and *Il‐6* in liver (h). Data are presented in mean ± SEM. Statistical differences were analyzed by two‐way ANOVA followed by Bonferroni's post hoc test (* = *p* < 0.05)

Inflammation can relate to senescence and its senescence‐associated secretory phenotype (Yousefzadeh et al., 2020). Senescence marker *p16* mRNA expression in liver was significantly higher in vehicle‐treated *Ercc1* ^*Δ/−*^ than in the corresponding WT mice, and there was a trend of *p16* upregulation in aorta (*p* = 0.07) (Figure 5a and b). *p21* mRNA expression was significantly higher in liver and aorta of vehicle‐treated *Ercc1* ^*Δ/−*^ than in the corresponding WT mice (Figure 5c and d). BAY 54–6544 treatment reduced *p16* and *p21* mRNA in *Ercc1* ^*Δ/−*^ mice, so that no significant increase vs. WT mice was present anymore (Figures 5a–d). The same was true for *Ccl2* and *Il‐6* mRNA expression in liver and aorta (Figures 5e–h).

**FIGURE 6:**
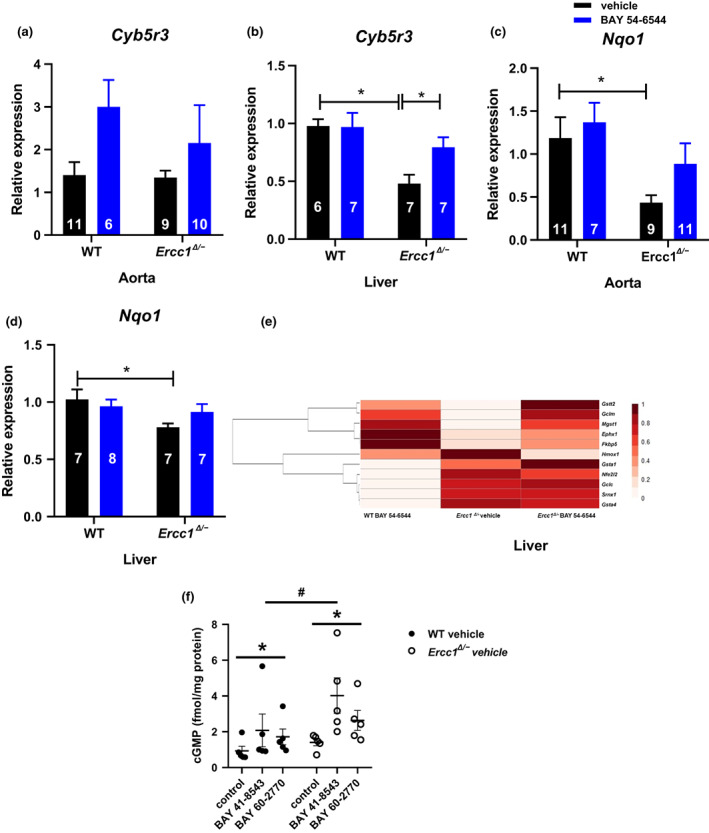
Gene expression of WT and *Ercc1* ^*Δ/−*^ mice treated either with vehicle or BAY 54–6544 for *Cyb5r3* in aorta (a) and liver (b), and for *Nqo1* in aorta (c) and liver (d). Data are presented in mean ± SEM. Statistical differences were analyzed by two‐way ANOVA followed by Bonferroni's post hoc test (* = *p* < 0.05). Heatmap of fold changes in mRNA expression of key antioxidant defense genes in liver of WT BAY 54–6544, *Ercc1* ^*Δ/−*^ vehicle and *Ercc1* ^*Δ/−*^ BAY 54–6544 treated mice against WT vehicle‐treated group. The intensity of color from light to dark shows an increasing pattern of expression. Hierarchical clustering on liver antioxidant genes was performed using Pearson correlation. (e). cGMP production levels in liver tissue from vehicle‐treated WT vs. *Ercc1* ^*Δ/−*^ mice when given ex vivo BAY 41–8543 and BAY 60–2770 for 30 minutes in organ baths in the presence of 10^−4^ Mol/L IBMX (f); data are presented in mean ± SEM. Statistical differences were analyzed by, Friedman test; interaction genotype x treatment: P = NS, two‐way ANOVA

Ageing has also been related to oxidative stress, and an upregulation of anti‐oxidant pathways has been identified as vascular protective and anti‐ageing. Two key reductase enzymes CYB5R3 and NADPH Quinone Dehydrogenase 1 (NQO1*)* are markers for anti‐oxidative effects of drug treatment, as well as dietary restriction (DR), the best‐known anti‐ageing treatment (Calvo‐Rubio et al., 2016; Diaz‐Ruiz et al., 2018). Moreover, CYB5R3 has been implicated in sGC reduction. *Cyb5r3* mRNA expression in aorta was at the same level in vehicle‐treated *Ercc1* ^*Δ/−*^ and the corresponding WT mice. After BAY 54–6544 treatment, there was a trend toward upregulation in *Ercc1* ^*Δ/−*^ and WT mice (Figure 6a). In contrast to vascular tissue, *Cyb5r3* mRNA expression was significantly downregulated in liver of vehicle‐treated *Ercc1* ^*Δ/−*^ compared with the corresponding WT mice. BAY 54–6544 treatment increased *Cyb5r3* in the *Ercc1* ^*Δ/−*^ mouse liver (Figure 6b).

*Nqo1* mRNA expression in both aorta and liver were significantly lower in vehicle‐treated *Ercc1* ^*Δ/−*^ than in the corresponding WT mice, and after treatment with BAY 54–6544, these differences were not significant anymore (Figure 6c and d). There were no differences within WT groups for *Cyb5r3* and *Nqo1* neither in aorta nor in liver (Figures 6a–d).

With the use of a set of key antioxidant genes that mark the protective effects of DR in ageing (Birkisdottir et al., 2021; Vermeij et al., 2016), we further mapped the effect of chronic BAY 54–6544 on anti‐oxidant pathways in liver. *Ercc1* ^*Δ/−*^ mice showed upregulated anti‐oxidant systems (Figure 6e). On top of that BAY 54–6544 further enlarged the panel of genes that show increased expression. The same panel was augmented in BAY 54–6544 treated WT mice (Figure 6e). As was the case in the vascular tissue, acute effects of BAY 41–8543 and BAY 60–2770 were similar, leaving no indication for domination of heme‐oxidated vs. native sGC or vice versa (Figure 6f).

## 3 DISCUSSION

This study investigated the potential role of sGC inactivation and reactivation with an sGC activator in non‐atherosclerotic vascular ageing inflicted by the DNA damage response (Vermeij et al., 2016), a major causal factor in ageing. We, therefore, tested the effect of chronic sGC activation in *Ercc1* ^*∆/−*^ accelerated ageing mice, focusing on changes of NO‐cGMP responsiveness, DNA damage‐related vascular dysfunction, and potential beneficial effects on survival. The beneficial effect of sGC activators on survival has been observed in cardiovascular disease models (Hahn et al., 2021; Hoffmann, Kretschmer, et al., 2015). In this study, we observed a significant improved survival rate in BAY 54–6544 treated *Ercc1* ^*∆/−*^ versus the vehicle‐treated ones (Figure 1). We applied a non‐pressor dosage of BAY 54–6544 (Figure 3). In a previous study, we showed that BP lowering with losartan had no effect on vascular dysfunction in *Ercc1* ^*∆/−*^ mice (Wu et al., 2017), whereas the pro‐survival effect of dietary restriction is associated with improved function of multiple organs and of vascular function (Vermeij et al., 2016). Combined, these observations plead for a blood pressure‐independent effect of BAY 54–6544. A question arising from the observation that BAY 54–6544 mimics aspect of dietary restriction is the possibility that the mice ate less. However, food intake was measured in our pilot experiment, and there was no indication of a reduced eating behavior after BAY 54–6544 (data not shown), nor did treatment animals lose weight compared with controls (Figure 1). BAY 54–6544 treatment improved reactive hyperemia was improved in *Ercc1* ^*∆/−*^ mice. After BAY 54–6544 treatment, a trend toward improved endothelium‐dependent response and a restored EDH pathway was observed in ex vivo vascular function measurements in *Ercc1* ^*∆/−*^ mice aorta. The endothelium‐independent response to NO, provided by SNP in organ baths, was significantly improved by BAY 54–6544 in *Ercc1* ^*Δ/−*^ aortas. Considering dysfunctional NO‐sGC‐cGMP signaling in *Ercc1* ^*∆/−*^ mice and the possible contribution of oxidative stress in impairing physiologic sGC function through oxidation, we assumed that the sGC activator is more efficacious than the stimulator. This prompted us to choose an sGC activator for the chronic treatment. However, to test whether the oxidative stress also directly affects sGC, the acute response of aortic rings was investigated by ex vivo organ bath measurements. The sGC activator is working preferentially on the oxidized and heme‐free sGC and the sGC stimulator targeting the native sGC. We observed the same maximum relaxation response for both sGC activator BAY 60–2770 and sGC stimulator BAY 41–8543 suggesting that there might be a balance of oxidized and native form of sGC in this acute setting. A similar observation was made when comparing cGMP production in liver between both BAY compounds. Therefore, heme oxidation of sGC does not appear to be central in failing NO—cGMP signalling in *Ercc1* ^*Δ/−*^ mice. This is in line with previous studies where it was shown that increased cGMP metabolism by phosphodiesterase was the major reason for decreased vasodilation (Bautista Nino et al., 2015; Durik et al., 2012). In parallel with the survival and vascular effect, we detected an increased expression of genes involved in anti‐oxidant defense, observed also in DR effects on ageing (Vermeij et al., 2016). In addition, a trend toward attenuation of inflammation and senescence markers was seen. These changes are not restricted to vascular tissue, but manifest themselves also in liver, an organ that is often used to study changes in common ageing pathways in various models (Schumacher et al., 2008; Vermeij et al., 2016).

### 3.1 Vasomotor signaling effects

#### 3.1.1 Acute sGC activation experiments

Vasodilator responses to BAY 41–8543 and BAY 60–2770 were similar in untreated *Ercc1* ^*Δ/−*^ vs. WT mice, and therefore heme‐oxidation did not seem to play a role. We previously found increased phosphodiesterase (PDE) metabolism of cGMP to be responsible for decreased NO—cGMP signaling in *Ercc1* ^*Δ/−*^ vascular tissue, and treatment with inhibitors thereof to exert beneficial vascular effects (Golshiri, Ataabadi, et al., 2021; Golshiri, Ataei Ataabadi, Brandt, et al., 2020; Golshiri, Ataei Ataabadi, et al., 2021). Our present results in aortic tissue organ bath experiments show that acute stimulation with BAY 41–8543 and BAY 60–2770 overcomes the phosphodiesterase increase and leads to up to 100% relaxation of the preconstriction with U46619 vs. 60–80% relaxation with SNP. This observation, however, prompts the question why SNP cannot elicit a maximal relaxation. A possible explanation might be the fact that NO given in high concentrations, which would occur with the administration of external NO donors such as SNP, can mitigate sGC activation through nitrosylation of cysteine residues in various regions of the enzyme, thus limiting its own cGMP‐generating capacity, putatively as a negative feedback mechanism (Beuve, 2017). Stimulation with ACh might not lead to such high NO levels, especially not in *Ercc1* ^*Δ/−*^ mice in which endothelial NO release is diminished (Durik et al., 2012). In summary, the following model can be constructed: In untreated WT mice, in which endothelium is intact, only sGC nitrosylation limits NO‐cGMP signaling. In contrast, in untreated *Ercc1* ^*Δ/−*^ mice ACh responses are decreased by lower endothelial NO release and higher PDE metabolism of cGMP in VSMC. SNP responses are limited by PDE and sGC nitrosylation in VSMC. Since BAY compounds bypass NO and sGC nitrolysation, these two limiting factors will not affect vasodilation responses to such compounds. In addition, the PDE increase is overruled. This might be due to abundant cGMP generation, saturating the pool of PDE with substrate.

#### 3.1.2 Chronic sGC activation studies

Chronic in vivo treatment with the sGC activator BAY 54–6544 in *Ercc1* ^*Δ/−*^ significantly improved the endothelium‐independent response to NO, provided by SNP, in conduit arteries measured ex vivo. In addition, there was a trend toward an improved endothelium‐dependent response to ACh after BAY 54–6544. Counterintuitively, instead of increasing NO‐dependent endothelium‐mediated relaxation chronic BAY 54–6544 increased the contribution of EDH in *Ercc1* ^*Δ/−*^. This paradoxical treatment effect can be explained by a paradigm that combines two signaling changes. The first change is induced by the decreased production of NO in the endothelium. Decreased NO‐mediated responses are often associated with increased EDH (Goto & Kitazono, 2019). As in *Ercc1* ^*Δ/−*^ mice NO production in the endothelium is reduced (Durik et al., 2012), an increase of EDH can be expected, causing the ACh response to rely more on EDH than on NO release as compared with WT mice. The present study shows that endothelial NO is not improved by chronic BAY 54–6544, and therefore EDH is expected to still be increased also after treatment in *Ercc1* ^*Δ/−*^ mice. The second signaling change is due to the impact of BAY 54‐6544‐induced cGMP increase in VSMC on EDH. Prolonged, strong increase of cGMP has been linked to dimerization of the effector protein kinase G (PKG). In turn, PKG dimerization has been shown to activate of BK_Ca2+_ channels in VSMC, a process that can also take place independently from cGMP through redox regulation and PKG oxidation (Ataei Ataabadi et al., 2020; Burgoyne et al., 2012; Feelisch et al., 2020; Jüttner & Roks, 2022; Sheehe et al., 2018). EDH involves an intricate interplay between endothelial and VSMC K^+^ channels (Goto & Kitazono, 2019), which can bring these two signaling changes together. TRAM34/Apamin‐sensitive EDH, the EDH tested in the present study, depends on generation of hyperpolarization in endothelial cells via IK_Ca2+_ and SK_Ca2+_ channels and the subsequent propagation of this hyperpolarization to VSMC, which can involve various VSMC K^+^ channels, one of which is the BK_Ca2+_ channel (Goto & Kitazono, 2019). Thus, increased EDH contribution to ACh in treated *Ercc1* ^*Δ/−*^ mice could be the sum of EDH substituting NO in the endothelium, and VSMC being more responsive to EDH via cGMP‐induced BK_Ca2+_ channels activation. In treated WT, where the emphasis is on endothelial NO instead of EDH generation, this would not be observed. Our results therefore warrant further interrogation of the involvement of EDH in vasomotor responses after chronic sGC activation in various models of decreased NO production.

The effects of sGC activation were not dependent of blood pressure. BP in *Ercc1* ^*Δ/−*^ mice was not significantly increased despite a clear decrease of vasodilation. In previous publications, we observed either significantly increased BP (Durik et al., 2012), or a non‐significant trend (K. Golshiri, Ataei Ataabadi, Portilla Fernandez, et al., 2020; Wu et al., 2017). Endothelial dysfunction may occur as a consequence of high blood pressure or be a causative factor itself for high blood pressure. If not fully accompanied by high BP at this stage, this may occur at a later stage, suggesting that high BP develops after endothelial dysfunction has occurred. Alternatively, other mechanisms may have resulted in BP lowering. BP increase may be attenuated by modulation of cardiac function, as previously demonstrated in an endothelial‐specific *Ercc1* knockout model (Bautista‐Nino et al., 2020).

### 3.2 Effects related to oxidative pathways

The acute ex vivo response of aortic rings and liver to the sGC activator BAY 60–2770 and to the sGC stimulator BAY 41–8543 was similar in all mouse groups, suggesting that neither native nor heme oxidation‐inactivated sGC was dominant in *Ercc1* ^*Δ/−*^ or WT mice. In addition, the maximal effect of both BAY compounds reached 100% of the preconstriction. Vascular expression of *Cyb5r3*, an anti‐oxidant enzyme that counters sGC oxidation, did not differ between *Ercc1* ^*Δ/−*^ and WT. This indicates that native and heme‐oxidized sGC are abundantly present in VSMC, and that activation of an anti‐oxidant defense response may not be necessary at this point. This might also be the case for liver tissue despite the reduced Cyb5r3 in *Ercc1* ^*Δ/−*^ this tissue. Previous results also showed no difference in sGC expression in aortic tissue between WT and *Ercc1* ^*Δ/−*^ mice (Durik et al., 2012).

Previously, we showed in *Ercc1* ^*Δ/−*^ mice and in mice with deletion of *Ercc1* in smooth muscle cells (SMC‐KO) that oxidative stress limited the responses to NO (Ehsan Ataei Ataabadi et al., 2021; Durik et al., 2012). We now show that heme‐oxidation of sGC does not contribute to this limitation. The present study was not conducted and designed to evaluate effects of oxidative stress in a broader context than sGC alone. Therefore, we cannot exclude that the increase of *Cyb5r3, Nqo1*, and other anti‐oxidant pathways after chronic BAY 54–6544 treatment, which were investigated in relation to survival pathways, contributed to the improved SNP responses.

### 3.3 Effects on survival and ageing pathways

Beneficial effect of sGC activators on survival has been observed in cardiovascular disease models such as Dahl salt‐sensitive rats (Hoffmann, Kretschmer, et al., 2015) and L‐NAME‐treated renin transgenic rats (Hahn et al., 2021). Furthermore, we have observed shortened life span in vascular‐specific *Ercc1* knockout mice, in which NO—cGMP signaling is prominently disturbed (Ehsan Ataei Ataabadi et al., 2021; Bautista‐Nino et al., 2020). Therefore, we were interested if sGC activator treatment in *Ercc1* ^*Δ/−*^ mice could have an impact on survival rates. In fact, the sGC activator could completely prevent the mortality of *Ercc1* ^*Δ/−*^ mice in the observation period. The underlying mechanism of these significant effect is not completely clear. The shortened lifespan of *Ercc1* ^*Δ/−*^ mice is mainly attributed to liver problems (McWhir et al., 1993; Selfridge et al., 2001). For this reason, liver is often chosen as a read‐out tissue to explore survival pathways in *Ercc1* ^*Δ/−*^ mice (Birkisdottir et al., 2021; Vermeij et al., 2016) and was also our motivation for the present study. Of note, beneficial effects of sGC activators on liver dysfunction have been observed before (Flores‐Costa et al., 2020; Hirth‐Dietrich et al., 2005; Sandner et al., 2017). Nevertheless, our results warrant future functional studies in multiple organs.

Up to now, DR is the only intervention that has been found effective in improving the survival rate in *Ercc1* ^*∆/−*^ mice (Vermeij et al., 2016). Shortening of lifespan in *Ercc1* ^*Δ/−*^ mice is resistant to rapamycin and anti‐oxidant therapy, which have shown effectiveness in various other animal models, and thus far was attenuated only by DR (Birkisdottir et al., 2021; Vermeij et al., 2016). When comparing effects of DR and sGC activator treatment in *Ercc1* ^*Δ/−*^ mice, we observe similar results on activation of anti‐oxidant genes. Also, the data hint toward attenuation of senescence inflammation markers, but not to the intensity level of dietary restriction. The findings are also reminiscent of earlier observations that indicated that cGMP signaling has favorable effects on inflammation and metabolism in adipose tissue of obese mice (Hoffmann, Etzrodt, et al., 2015; Sanyal et al., 2017). Interestingly, the observation that the survival effect of sGC activation coincides with increase of CYB5R3 and NQO1 expression is in striking parallel with the finding that overexpression of both these NADH‐dehydrogenases leads to a modest increase in lifespan, better physical performance, and decreased chronic inflammation in ageing mice (Diaz‐Ruiz et al., 2018). Also, DR is accompanied by the rescue of age‐associated decline of CYB5R3 and NQO1 function (de Cabo et al., 2009; Hyun et al., 2006). Although the significance of the observation remains to be further established, our observation that the increase in the senescence and inflammation markers is attenuated is in further agreement with a pro‐survival effect of BAY 54–6544. The idea that senescence and inflammation are connected in regulation of life‐ and health‐span has been demonstrated in several normal ageing and DNA damage‐induced accelerated ageing rodent models and has led to the development of senolytic drugs, which are not yet applied in the clinic (Baar et al., 2017; Baker et al., 2016; Baker et al., 2011; Tchkonia et al., 2013; Yousefzadeh, Henpita, et al., 2021; Yousefzadeh, Flores, et al., 2021; Yousefzadeh et al., 2020). In summary, the sGC activator treatment, in intervention mode that is currently applied in clinical treatment of pulmonary hypertension, appears to display similarities to DR, and perhaps of senolytics. Further examination of the value of this observation is warranted.

## 4 CONCLUSION

In summary, we could show that sGC activator treatment restores vasodilator dysfunction and improves longevity and ageing markers in mice that display accelerated ageing due to defective DNA repair. To the best of our knowledge, the current study is the first one showing positive effects of chronic sGC activator treatment on attenuating general ageing markers. The findings of this study may be used as the very first evidence that clinically applicable cGMP‐modulating drugs may prevent vascular ageing. This may have important implications for repurposing of such medicines, which are currently used only in an advanced state of ageing‐related cardiovascular disease. As shown here, their effects might not be limited to the cardiovascular system and further research characterizing the impact on health span is indicated.

## 5 MATERIALS AND METHODS

### 5.1 Pilot study

Before starting intervention experiments, we ran a dose‐finding study to choose the most appropriate dose of the sGC activator BAY 54–6544 based on blood‐pressure lowering efficacy. Fifteen WT animals were randomized into 3 groups of 5 animals and treated either with vehicle, low dose BAY 54–6544 (80 mg/kg/day) or high dose BAY 54–6544 (200 mg/kg/day). All chows supplemented with BAY 54–6544 and placebo chow were obtained from Bayer AG, Wuppertal, Germany. BP was measured with tail cuff in 5 consecutive days and the 5th day considered as the main measurement. The detailed protocol for tail cuff measurements is explained below. We aimed to choose a dose without or only minor effects on systemic blood pressure. In addition, we measured the daily amount of chow that was consumed to investigate the possible effect of BAY 54–6544 on food consumption.

### 5.2 Animals

*Ercc1* ^*Δ/−*^ and *Ercc1* ^+/+^ F1 mice had a hybrid C57BL6J: FVB background and were generated by cross‐breeding of parents with a pure C57BL6J and FVB background, as described before (Dollé et al., 2011). *Ercc1* ^*Δ/−*^ mice are hemizygous for a single truncated *Ercc1* allele, encoding a protein lacking the last seven amino acids in all body cells. The hybrid background of the experiment mice prevents strain‐specific phenotypes. Breeding was accomplished at the Erasmus MC animal facility. Mice were housed in individually ventilated cages in a controlled environment (20–22°C, 12 h light:12 h dark cycle), with access to food and water ad libitum. The animals were weighed and visually inspected every day by animal caretakers to warrant their well‐being. All animal procedures were accomplished at Erasmus MC facility for animal experiments followed by the guidelines from Directive 2010/63/EU of the European Parliament on the protection of animals used for scientific purposes. All animal studies were approved by the National Animal Care Committee and the local administration within Erasmus University Medical Center Rotterdam.

### 5.3 Study design

In total, 30 *Ercc1* ^*Δ/−*^ mice and 32 of their age‐matched WT littermates (*Ercc1* ^*+/+*^) at the age of 8 weeks were randomized into four groups: one group of *Ercc1* ^*Δ/−*^ mice and one WT group were given vehicle chow; another group of *Ercc1* ^*Δ/−*^ and WT mice received BAY 54–6544 (200 mg/kg/day) in chow for 8 weeks. Both male and female animals were used. BP and superficial blood flow were measured one week prior to sacrifice. At the age of 17 weeks, mice were sacrificed, tissues were collected and snap‐frozen, and wire myography measurements were performed.

### 5.4 Blood pressure measurement

Blood pressure (BP) was measured with a non‐invasive method in conscious mice using the tail cuff technique (CODA High‐Throughput device, Kent Scientific). BP was measured on 5 consecutive days and each session consisted of 30 measurement cycles per mouse. The first 4 days were performed as acclimatization sessions and the 5th day was the main measurement for each mouse. BP values are reported as the average of all valid cycles recorded at Day 5 (Durik et al., 2012).

### 5.5 Microvascular vasodilator function in vivo

We assessed in vivo vasodilator function using Laser Doppler perfusion imaging (LDPI, Perimed, PeriScan PIM 3 System). Reactive hyperemia, defined as the hindleg perfusion increase after temporary occlusion of the blood flow, was calculated. One day prior to laser doppler in the left hindleg, hair was removed by hair‐removal cream. The hindleg was kept still with the aid of a fixation device. After recording baseline perfusion for 5 min, blood flow was occluded for 2 min with a tourniquet. After releasing the tourniquet, blood flow was monitored for 10 min to observe its return to the post‐occlusion baseline and to record hyperemia. During all the measurements, mice were under 2.8% isoflurane anesthesia, and body temperature was kept at 36.4–37.0°C by means of a heating pad with rectal temperature probe feedback. For each mouse, the maximum flow response after occlusion and the AUC relative to the post‐occlusion baseline were calculated. Only the area above the baseline was considered and values below the baseline were set at 0.

### 5.6 Ex vivo vascular function assessment

Immediately after sacrificing the mice, thoracic aortas were carefully isolated from mice and kept in cold Krebs–Henseleit buffer (in mmol/L: NaCl 118, KCl 4.7, CaCl_2_ 2.5, MgSO_4_ 1.2, KH_2_PO_4_ 1.2, NaHCO_3_ 25, and glucose 8.3 in distilled water; pH 7.4). Vessel segments of 1.5–2 mm length were mounted in small wire myograph organ baths (Danish Myograph Technology, Aarhus, Denmark) containing 6 ml of Krebs–Henseleit buffer oxygenated with 95% O_2_ and 5% CO_2_ at 37°C. The tension was normalized by stretching the vessels in steps until 90% of the estimated diameter at which the effective transmural pressure of 100 mmHg is reached (Bridges et al., 2011). Thereafter, the viability of the vessels was checked by inducing contractions with 30 and 100 mmol/L KCl. After reaching the maximum contraction induced by KCl, vessels were washed 4 times with a 5‐minute interval. To evaluate vasodilatory responses, aortic segments were preconstricted with 30 nmol/L of U46619 (a thromboxane A2 analog) resulting in a preconstriction corresponding with 50–100% of the response to 100 mmol/L KCl.

After reaching a contraction plateau in response to U46619, CRCs were constructed with the endothelium‐dependent vasodilator acetylcholine (ACh) at cumulative doses (10^−9^–10^−5^ mol/L). To evaluate the involvement of the NO‐cGMP pathway, one segment was preincubated with N^G^‐nitro‐L‐arginine methyl ester salt (L‐NAME, 10^−4^ mol/L), an endothelial nitric oxide synthase inhibitor. To investigate the role of EDH, the small conductance Ca^2+^‐activated K^+^ channel inhibitor (SK_Ca2+)_ apamin (100 nmol/L) and the intermediate conductance Ca^2+^‐activated K^+^ channel (IK_Ca2+)_ inhibitor TRAM34 (10 μmol/L) were added on top of L‐NAME.

In parallel rings, after reaching a contraction plateau in response to U46619, endothelium‐independent CRCs to the NO‐donor SNP (10^−11^–10^−4^ mol/L) were constructed. Moreover, endothelium‐independent CRCs to the sGC activator BAY 60–2770 (10^−10^–10^−5^ mol/L) for all groups and endothelium‐independent CRCs to the sGC stimulator BAY 41–8543 (10^−10^–10^−5^ mol/L) for vehicle‐treated groups were constructed. To avoid bias by intrinsic release of NO, all segments were preincubated with L‐NAME 10^−4^ mol/L added 20 minutes before preconstriction.

### 5.7 Analysis of plasma cytokine levels

IL‐2, IL‐4, IL‐6, IFN‐γ, and TNF‐α were measured in mouse plasma using a high sensitivity ProcartaPlex 96 well for mouse Multiplex assay from Thermofisher (EPXS050‐22199‐901). Samples and standards were run according to the manufacturer instructions and analyzed with Bio‐Plex MAGPIX multiplex reader (Bio‐Rad: Laboratory Medical Immunology, Erasmus MC) (ULOQ/LLOQ detection limits are IFN‐γ: 260 / 0.06348, TNF‐α: 1390 / 0.34, IL‐2: 490 / 0.12, IL‐4: 560 / 0.14, IL‐6: 2850 / 0.70. All the concentrations are in pg/ml).

### 5.8 Quantitative real‐time PCR

Total RNA was isolated from aorta and liver of all groups of mice using the RNeasy Mini Kit (Qiagen). RNA concentration and quality were determined with a NanoDrop spectrophotometer (Isogen Life Science, IJsselstein, The Netherlands). RNA was reverse transcribed by the use of Quantitect Rev. Transcription Kit (Qiagen) according to the manufacturer's protocol. The cDNA was then amplified by quantitative real‐time PCR on a QuantStudio 7 Flex Real‐time PCR system (Applied Biosystems). Each reaction was performed in duplicate with SYBR Green PCR Master Mix (UK, Applied Biosystems). PCR cycling conditions were 50°C for 2 min, 95°C for 2 min, followed by 40 cycles of 95°C for 15 s, and 60°C for 1 min. *β‐actin* and *Hprt1* were used as household genes. Results from unreliable duplicates or melting curves were discarded. Gene expression changes measured by quantitative real‐time PCR displayed as relative expression to WT vehicle‐treated group. Fold changes of key antioxidant defense genes in liver of WT BAY 54–6544, *Ercc1* ^*Δ/−*^ vehicle and *Ercc1* ^*Δ/−*^ BAY 54–6544 treated mice were calculated against WT vehicle‐treated group and displayed in a heatmap. Genes amplified along with the used primer combinations are given in Supplementary Table 1.

### 5.9 Liver cGMP production assay

To investigate sGC activity in liver tissue, an adapted protocol of Calmasini et al. (2016) was used (Calmasini et al., 2016). Liver tissue was freshly obtained, diced in ~1 mm‐sized parts, and kept in aerated Kreb's buffer at 37°C in organ baths. After 30 min of acclimatization, 10 mM of 3‐isobutyl‐1‐methylxanthine (IBMX) was added to block cGMP degradation by phosphodiesterases. After 10 min of IBMX incubation, sGC stimulator BAY 41–8543 (300 nM), sGC activator BAY 60–2770 (300 nM) or vehicle (dimethylsulfoxide 0.1%) was added, thus creating 3 paired observations per mouse. After 30 min of incubation, the liver tissue was snap frozen in liquid nitrogen. The levels of cGMP were measured using an enzyme‐linked immunosorbent assay according to the manufacturer's descriptions (Enzo Life Sciences, Farmingdale, NY, USA).

### 5.10 Statistical methods

Data are presented as mean and standard error of the mean (mean ± SEM), unless otherwise indicated. Differences among the groups, depending on the number of variables, were analyzed either by one‐way ANOVA followed by Dunnett's post hoc test or two‐way ANOVA followed by Bonferroni's post hoc test. Survival rate differences were evaluated by log‐rank (Mantel‐Cox) test. Differences between CRCs were tested by general linear model for repeated measures (GLM‐RM) (sphericity assumed). Multiple comparisons of non‐normally distributed, paired data were tested with a Friedman test. *p* values below 0.05 were considered as significant.

## AUTHOR CONTRIBUTIONS

E.A.A., K.G., A.H.J.D., and A.R. involved in conceptualization. H.N.K., I.v.d.P., and R.d.V. involved in data curation. E.A.A., K.G., A.A.J., R.d.V., R.B., and A.R. contributed in formal analysis. A.R. involved in funding acquisition. E.A.A., K.G., A.A.J., R.d.V., F.P.J.L, N.M.A.N, H.N.K, I.V.D.B.G. and A.R. performed investigation. E.A.A., K.G., I.v.d.P., R.d.V., A.H.J.D., I.V.D.B.G, N.A.M.N., H.N.K. and A.R. performed methodology. E.A.A., K.G., R.B., I.v.d.P., R.d.V., and A.R. involved in project administration. P.S., A.H.J.D., and A.R. collected resources. P.S, A.H.J.D., and A.R. supervision. A.H.J.D. and A.R. involved in validation. E.A.A. performed visualization. E.A.A. and A.R. performed writing—original draft. K.G., A.A.J., I.v.d.P., W.A.D., P.S., A.H.J.D., and A.R. involved in writing—review and editing. All authors have read and agreed to the published version of the manuscript.

## CONFLICT OF INTEREST

Peter Sandner is employee of Bayer AG, Leverkusen, Germany.

## Supporting information


Table S1

## Data Availability

The data that support the findings of this study are available from the corresponding author upon reasonable request.
