# Supplementary material for: Soluble guanylate cyclase activator BAY 54–6544 improves vasomotor function and survival in an accelerated ageing mouse model
Source: Aging Cell. 2022 Aug 27;21(9):e13683. doi: 10.1111/acel.13683 (PMC9470884; doi:10.1111/acel.13683)
Supplement: Supplementary file 1 — Table S1 [file ACEL-21-e13683-s001.docx]

**Table S1**

| Gene | Sense | Antisense |
| --- | --- | --- |
| Mouse *Cyb5r3* | 5’-CCCGACATCAAGTACCCTCT-3′ | 5’-GCCATCGATCCTAGTCGAG-3′ |
| Mouse *Nqo1* | 5’-TTCTGTGGCTTCCAGGTCTT-3′ | 5’-AGGCTGCTTGGAGCAAAATA-3′ |
| Mouse *p16* | 5’-CGCTCTGGCTTTCGTGAACA-3′ | 5’-GTTGCCCATCATCATCACCTGG-3′ |
| Mouse *p21* | 5’-GTCAGGCTGGTCTGCCTCCG-3′ | 5’-CGGTCCCGTGGACAGTGAGCAG-3′ |
| Mouse *Ccl2* | 5’-AGCTGTAGTTTTTGTCACCAAGC-3′ | 5’-TGTCTGGACCCATTCCTTCTTG-3′ |
| Mouse *Il-6* | 5’-TCCAGTTGCCTTCTTGGGAC-3′ | 5’-GTGTAATTAAGCCTCCGACTTG-3′ |
| Mouse *Gsta1* | 5’-CTTCTGACCCCTTTCCCTCT-3’ | 5’-ATCCATGGGAGGCTTTCTCT-3’ |
| Mouse *Nfe2l2* | 5’-AGGACATGGAGCAAGTTTGG-3’ | 5’-TCTGTCAGTGTGGCTTCTGG-3’ |
| Mouse *Gstt2* | 5’-CGAGCAATTCTCCCAGGTGA-3’ | 5’-TATTCGTGGACTTGGGCACG-3’ |
| Mouse *Fkbp5* | 5’-TGTTCAAGAAGTTCGCAGAGC-3’ | 5’-CCTTCTTGCTCCCAGCTTT-3’ |
| Mouse *Srxn1* | 5’-TGAGCAGCTCCTCTGATGTG-3’ | 5’-GCTGAGGTGACAATTGACTATGG-3’ |
| Mouse *Gsta4* | 5’-TCGATGGGATGATGCTGAC-3’ | 5’-CATCTGCATACATGTCAATCCTG-3’ |
| Mouse *Gclm* | 5’-TGGAGCAGCTGTATCAGTGG-3’ | 5’-CAAAGGCAGTCAAATCTGGTG-3’ |
| Mouse *Hmox1* | 5’-CAGGTGATGCTGACAGAGGA-3’ | 5’-ATGGCATAAATTCCCACTGC-3’ |
| Mouse *Gclc* | 5’-AGATGATAGAACACGGGAGGAG-3’ | 5’-TGATCCTAAAGCGATTGTTCTTC-3’ |
| Mouse *Ephx1* | 5’-GAGTGGAGGAACTGCACACC-3’ | 5’-AGCACAGAAGCCAGGATGA-3’ |
| Mouse *Mgst1* | 5’-CTCGGCAGGACAACTTGC-3’ | 5’-CCATGCTTCCAATCTTGGTC-3’ |
| Mouse *β-actin* | 5’-TTCTTGGGTATGGAATCCTGTGG-3’ | 5’-GTCTTTACGGATGTCAACGTCAC-3’ |
| Mouse *Hprt1* | 5’-CCTAAGATGAGCGCAAGTTGAA-3’ | 5’-CCACAGGACTAGAACACCTGCTAA-3’ |
